# Enriched Monolayer Precursor Cell Cultures from Micro-Dissected Adult Mouse Dentate Gyrus Yield Functional Granule Cell-Like Neurons

**DOI:** 10.1371/journal.pone.0000388

**Published:** 2007-04-25

**Authors:** Harish Babu, Giselle Cheung, Helmut Kettenmann, Theo D. Palmer, Gerd Kempermann

**Affiliations:** 1 Max Delbrück Center for Molecular Medicine (MDC) Berlin-Buch, Berlin, Germany; 2 VolkswagenStiftung Research Group, Department of Experimental Neurology, Charité University Medicine Berlin, Berlin, Germany; 3 Department of Neurosurgery, Stanford University, Palo Alto, United States of America; University of Sydney, Australia

## Abstract

**Background:**

Stem cell cultures are key tools of basic and applied research in Regenerative Medicine. In the adult mammalian brain, lifelong neurogenesis originating from local precursor cells occurs in the neurogenic regions of the hippocampal dentate gyrus. Despite widespread interest in adult hippocampal neurogenesis and the use of mouse models to study it, no protocol existed for adult murine long-term precursor cell cultures with hippocampus-specific differentiation potential.

**Methodology/Principal Findings:**

We describe a new strategy to obtain serum-free monolayer cultures of neural precursor cells from microdissected dentate gyrus of adult mice. Neurons generated from these adherent hippocampal precursor cell cultures expressed the characteristic markers like transcription factor Prox1 and showed the TTX-sensitive sodium currents of mature granule cells in vivo. Similar to granule cells in vivo, treatment with kainic acid or brain derived neurotrophic factor (BDNF) elicited the expression of GABAergic markers, further supporting the correspondence between the in vitro and in vivo phenotype. When plated as single cells (in individual wells) or at lowest density for two to three consecutive generations, a subset of the cells showed self-renewal and gave rise to cells with properties of neurons, astrocytes and oligodendrocytes. The precursor cell fate was sensitive to culture conditions with their phenotype highly influenced by factors within the media (sonic hedgehog, BMP, LIF) and externally applied growth factors (EGF, FGF2, BDNF, and NT3).

**Conclusions/Significance:**

We report the conditions required to generate adult murine dentate gyrus precursor cell cultures and to analyze functional properties of precursor cells and their differentiated granule cell-like progeny in vitro.

## Introduction

Despite the fact that the majority of studies on adult neurogenesis and adult neural precursor cells are done in mice, no protocol for long-term monolayer stem cell cultures from the adult murine dentate gyrus has been reported. Precursor cells of the adult dentate gyrus proliferate in the subgranular zone (SGZ) and generate granule cells that are added to the adjacent granule cell layer [1], [2]. The precursor cells are found within a highly specific stem cell niche. Neuronal development from these precursor cells is regulated in an activity-dependent way and multiple regulatory mechanisms reach the developing cells. Neuronal development in the adult means the realization of the genetic potential of the precursor cells in close guiding interaction with the local permissive microenvironment. As yet it remains unknown how far the in vivo neurogenic niche could be recapitulated ex vivo.

Monolayer cultures have been established only for rats and cultures from mouse hippocampus have only been maintained as so-called neurosphere cultures derived from the entire hippocampal formation [3], [4], [5]. Consequently, information about stem cells in the adult mouse dentate gyrus has been either inferred from experiment in rats or from mouse neurosphere studies that have not assessed how well the model system produced cells that mirrored the in vivo situation. Such validation would be important for using precursor cells from the adult hippocampus as relevant model system for cell-based plasticity in the adult hippocampus and also for the future application of stem cells in replacing damaged or lost neurons in the adult and aging brain [6]. To realize the potential of stem cell therapy, it is vital that the exact phenotype of the neurons that should be replaced can be generated. Dentate gyrus granule cells might not be the prime targets for ex vivo cell replacement therapy but using hippocampal precursor cell cultures as valid model system for the regulatory mechanisms of cellular plasticity in the adult brain requires that the cultures yield cells with properties truly reflecting the neuronal phenotype of granule cells in vivo.

Neurospheres, which are the most widely used form of culturing mouse neural precursor cells, have limitations because they are heterogeneous [7]. Cells at the core of the sphere differentiate and each neurosphere might actually contain only very few precursor cells which become diluted with each ensuing passage [7]. Functional studies requiring real-time resolution on a single cell level are limited, if not impossible, in the multicellular clusters. Adherent monolayer cultures better reveal the morphology of individual cells and fully expose cells to a controlled extracellular environment. Monolayer cultures were originally described for hippocampal stem cells from adult rats or from cells isolated from whole mouse brains but have not yet been adapted for adult mouse hippocampus or subdissected hippocampal dentate gyrus [8], [9], [10]. Furthermore, it has been disputed whether stem cells in the strict sense of the definition exist in the adult hippocampus [4], [5]. An adequate hippocampal stem cell culture model would have to meet two key requirements: the culture would have to build on a reliable expanding precursor cell population with known “stemness” properties and generate the appropriate neuronal phenotype of dentate gyrus granule cells. Here we have developed a murine monolayer stem cell culture system to directly test this hypothesis.

We use the following nomenclature: “stem cells” are cells with demonstrated self-renewal (the ability to generate additional stem cells) and multipotency (the ability to generate neurons, oligodendrocytes and astrocytes); progenitor cells are the progeny of stem cells with limited self-renewal and lineage restriction. “Precursor cells” serves an umbrella term encompassing stem and progenitor cells as well as cells with undetermined but assumed degrees of stemness.

We report that adult neural precursor cell culture can be established from microdissected dentate gyrus and can be propagated for many passages to provide an inexhaustible source of hippocampal precursor cells ex vivo. The neurons that could be derived from these precursors cells showed many properties of dentate gyrus granule cells. The proliferating precursor cells in contrast revealed features of radial glia. The precursor cells showed features of self renewal when plated as single cells and under differentiation conditions generated cells with in vitro properties of neurons and glia.

## Results

### Isolation and characterization of proliferating precursor cells from micro-dissected adult murine dentate gyrus

**Figure 1:**
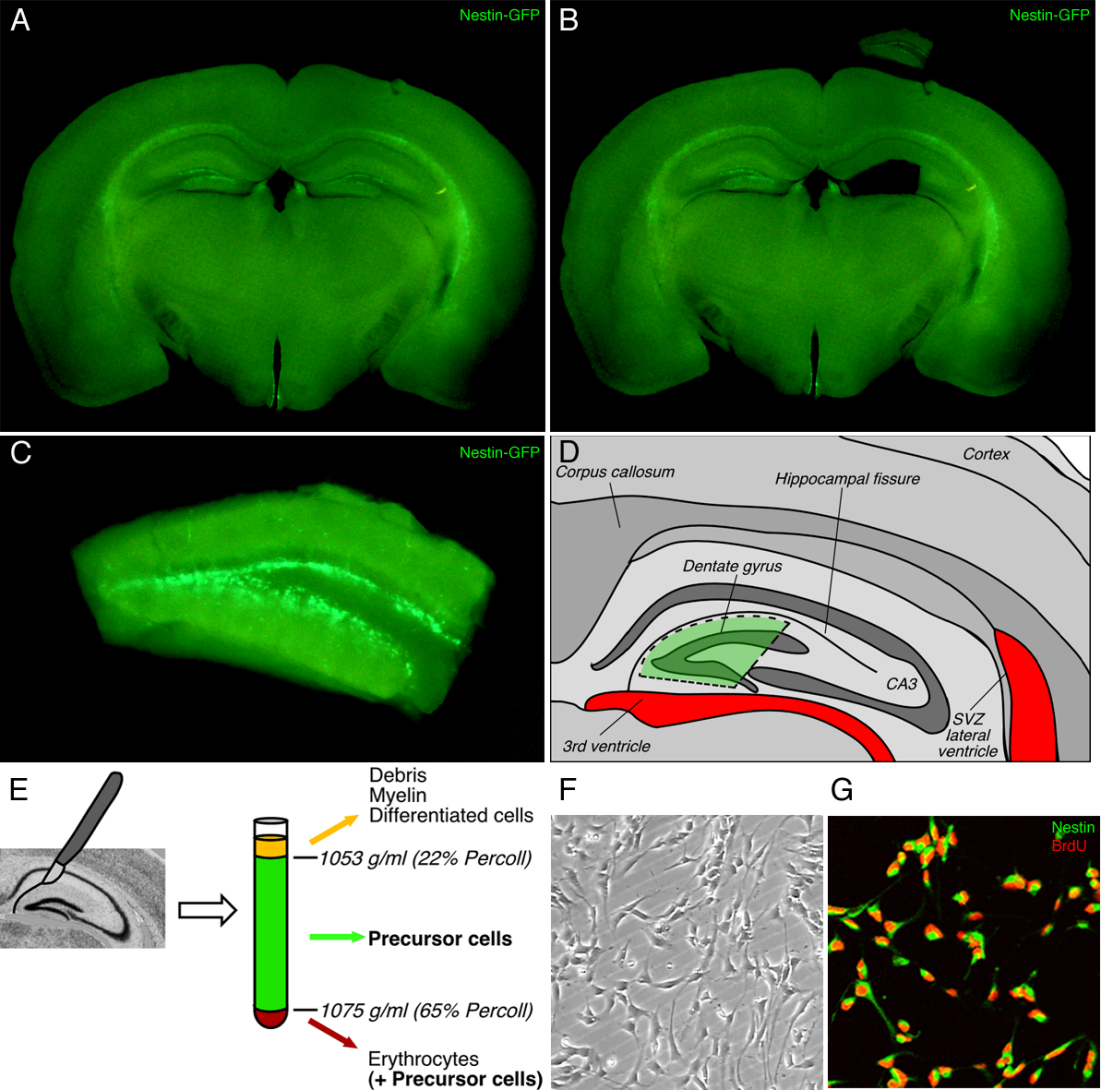
Isolation of endogenous precursor cells from the adult mouse dentate gyrus. A; Coronal 300 µm vibratome section through the brain of a Nestin-GFP transgenic mouse. B; As a second step, the dentate gyrus was isolated from the rest of the tissue by microdissection. The dissected dentate gyrus is shown at the top. C; The dissected dentate gyrus, as depicted in B, was further trimmed by placing two cuts that separate the dentate gyrus from ventricular tissue (of the 3^rd^ ventricle) and area CA3 (see also D). Nestin-GFP expression in the subgranular zone is seen with high concentration at the medial tip of the granule cell layer. D; Schematic drawing depicting the dissected region that was used for isolation of the precursor cells. The localization of the potentially contaminating ventricular areas is highlighted in red. The dashed lines indicate the cuts described in C. E; The tissue was homogenized and separated on a Percoll density gradient. Proliferating precursors were present in the region with density>1.053 gm/ml (>22% Percoll). The Erythrocytes were enriched in the region with density>1.075 gm/ml (>65% Percoll). F; phase contrast image of mouse precursor cells grown as adherent cultures. Note the phase bright appearance that is characteristic of proliferating precursor cells. G; Incubation with BrdU (20 µM) revealed that isolated nestin-expressing cells were proliferating. Nestin (Green), BrdU (Red).

Dissection of the entire hippocampus to initiate hippocampal precursor cell cultures had previously led to controversy because corpus callosum and the subventricular zone (SVZ) of the lateral ventricles are potential sources of contamination with other stem cells [4]. We therefore used standardized microdissection procedures to separate the dentate gyrus from other hippocampal regions along the hippocampal fissure (Fig. 1a–c). The brains were sliced using a vibratome and the dentate gyrus dissected from the rest of the hippocampus and the adjacent tissue under a microscope. Particular care was taken to avoid any contamination by tissue from the SVZ of the lateral ventricles and also the 3^rd^ ventricle. Within each slice, a cut was placed between the dentate gyrus and the ventricular wall. The thickness of the slice of only 300 µm allowed to place this cut without the danger of contamination due to the curved morphology of the ventricle and the dentate gyrus in deeper layers below the visible superficial plane. In any case, the 3^rd^ ventricle contains nestin-positive cells but, in contrast to the SVZ of the lateral ventricles, only very few proliferating cells [11]. Possibly, 3^rd^ ventricle precursor cells give rise to hypothalamic cells [12], [13], [14].

In addition, we used mice expressing green fluorescent protein (GFP) under the neural enhancer element of the nestin promoter [15] (Fig. 1a). Within the dissected area, GFP-expressing cells were present only in the region between hilus and granule cells layer or SGZ (Fig. 1c). Thus, if analyzed immediately after isolation, any nestin-GFP expressing cells in the cultures could only originate from this region. We also took care to include the tip of the dentate gyrus, the area of the hippocampus with the highest density of proliferating cells.

The tissue was homogenized and digested with papain. We carried out a series of continuous and step Percoll gradients to isolate the proliferating progenitor from other cells in the homogenate. Proliferating precursor cells have a high density, allowing these less buoyant cells to be separated from the non-proliferating cells on continuous density gradients spanning between 1.053–1.075 g/ml and on centrifugation at>10,000×g. Centrifugation yielded three visible cell layers (Fig. 1e). The uppermost layer (1.053 g/ml) was composed of cell debris, myelin and differentiated cells. The lowermost layer (>1.075 gm/ml) contained erythrocytes. The middle layer was collected and used to initiate cultures (Fig. 1e).

Immunohistochemistry for nestin, glial fibrillary acidic protein (GFAP) and polysialylated neural cell adhesion molecule (PSA-NCAM), markers of hippocampal precursor cells in vivo [16], [17], did not reveal putative precursor cells below 1.053 g/ml. The fraction>1.075 gm/ml contained cells expressing nestin and PSA-NCAM. Of all the nucleated cells recovered from>1.053 g/ml 23.5±1.7% were nestin-positive (mean±s.e.m.), 42.9±4.6% were positive for GFAP, and 28.0±6.00% of the nestin-positive cells were PSA-NCAM-positive.

Cells were cultured on poly-D-lysine and laminin-coated surfaces in defined medium with mitogens fibroblast growth factor (FGF2) and epidermal growth factor (EGF). After a few days, the cells began to proliferate and populate the culture dish (Fig. 1f). Upon passaging the cells did not persist in FGF2 alone, but required both EGF and FGF2 for continued proliferation.

Cells were passaged every 5–6 days, when the cells reached 80–90% confluency. We found that cell growth and survival in culture were generally highly dependent on cell density. Optimal growth required that cultures maintained a minimum density at plating. Failure to passage the cells in a timely manner (despite medium change) resulted in massive cell death, suggesting that higher cell densities negatively affected stem cell survival even in the presence of trophic support. In addition to the macroscopic observation that the cells were increasing in numbers, we examined whether the cells were indeed proliferating. We incubated the cells with permanent S-phase marker bromodeoxyuridine (BrdU) and subsequent immunohistochemical detection of BrdU. This revealed that up to 95% of the cells were in cell cycle (Fig. 1g). BrdU-positive cells had the spindle shaped morphologies characteristic of precursor cells (Fig. 1g), whereas BrdU-negative cells showed flat morphologies resembling mature astrocytes. Almost all BrdU-positive cells were also nestin-positive.

The cells could be passaged up to 65 times, beyond which no attempts were undertaken. Proliferating cells could be frozen down in 10% DMSO in proliferation medium and be recovered by rapidly bringing them to 37°C and plating in fresh medium (not shown).

### Proliferating precursor cells exhibit radial glia-like properties and differentiate into neurons

**Figure 2:**
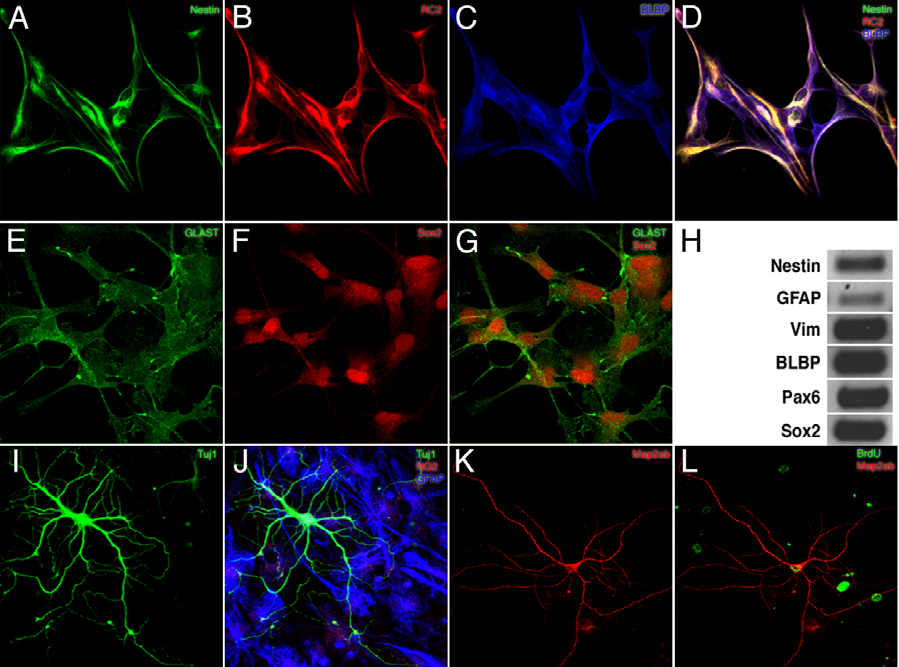
Proliferating cells in culture showed features of radial glia and upon differentiation generated neurons and astroglial cells. A–D; Proliferating cultures expressed Nestin (A), RC2 (B), BLBP (C) D; Overlay of the three markers Nestin (green), RC2 (red) and BLBP (blue). E–G; Precursor cells also immunostained for GLAST (E) and Sox2 (F), overlay of GLAST and Sox2 (G). H; RT-PCR analysis of mRNA from precursor cells further corroborated the suggestive radial glia-like phenotype: Nestin, GFAP, Vimentin, BLBP, Pax6, Sox2. I,J; Under differentiation conditions precursor cells generated neurons and astroglia (bIII-tubulin (green) GFAP (blue) and NG2 (oligodendrocyte precursors). K,L; Addition of BrdU before the transfer to differentiation conditions revealed BrdU (green) in neurons expressing Map2ab (red), confirming that the neuron-like cells originated from a proliferating precursor cell.

Precursors cells in the adult SGZ show features of radial glia [18], [19]. We thus examined whether precursor cells from the adult dentate gyrus had radial glia-like features in culture. We detected features of radial glia indicated by immunoreactions with antibodies against RC2 antigen [20] and brain lipid binding protein (BLBP; Fig. 2a–d;). The cells also showed immunoreaction with antibodies against GLAST and Sox2 (Fig 2e–g). In addition, the cells expressed mRNA of nestin, GFAP, Sox2, Pax6, and vimentin (Fig 2h). This set of markers is diagnostic for radial glia. BLBP and Sox-2 are also expressed by proliferating cells in the SGZ [19]. The expression of markers that are also expressed by radial glia is obviously not yet identical to being radial glia (hence our designation as radial glia-like) but the bottom line remains that ex vivo the precursor cells maintained characteristic features of their presumed in vivo counterparts.

In the course of the differentiation process, the undifferentiated precursor cells started as polygonal to spindle-shaped cells, subsequently acquiring bipolar morphologies. At 10–14 days, the neurons began to develop multiple elaborate processes that were positive for β-III-tubulin and typical for mature neurons (Fig. 2i,j). BrdU incubation and subsequent differentiation yielded neurons positive for microtubule associated protein 2ab (Map2ab) and BrdU-labeled nucleus (Fig. 2k,l). This indicated that proliferating precursor cells had been induced to differentiate into neurons. Cells lacking neuronal markers were strongly positive for GFAP and morphologically resembled protoplasmic or type-II astrocytes (Fig. 2j).

Quantitatively, the generation of neurons was dependent on both cell density and the presence of external soluble factors. We empirically found that the best density for neuronal lineage commitment was 1-2×10^4^ cells/cm^2^. The proportion of cells adopting neuronal fates (0.8–1%) was far below the rate seen in the neurogenic area of the dentate gyrus in vivo, where about 80% of the new cells are neurons [21] but is consistent with the level of in vitro neurogenesis from adult neural stem cells in prior reports [22]

Further studies are needed to ascertain the combinatorial patterns of stimulation that direct neural precursor cells to a particular lineage. For example, the influences of endothelial cells or astrocytes provide important neurogenic cues but the exact signaling molecules that promote neurogenesis in this context are still not well defined [23], [24], [25].

### Adult-derived hippocampal precursor cells differentiate into neurons with granule cell-like properties

**Figure 3:**
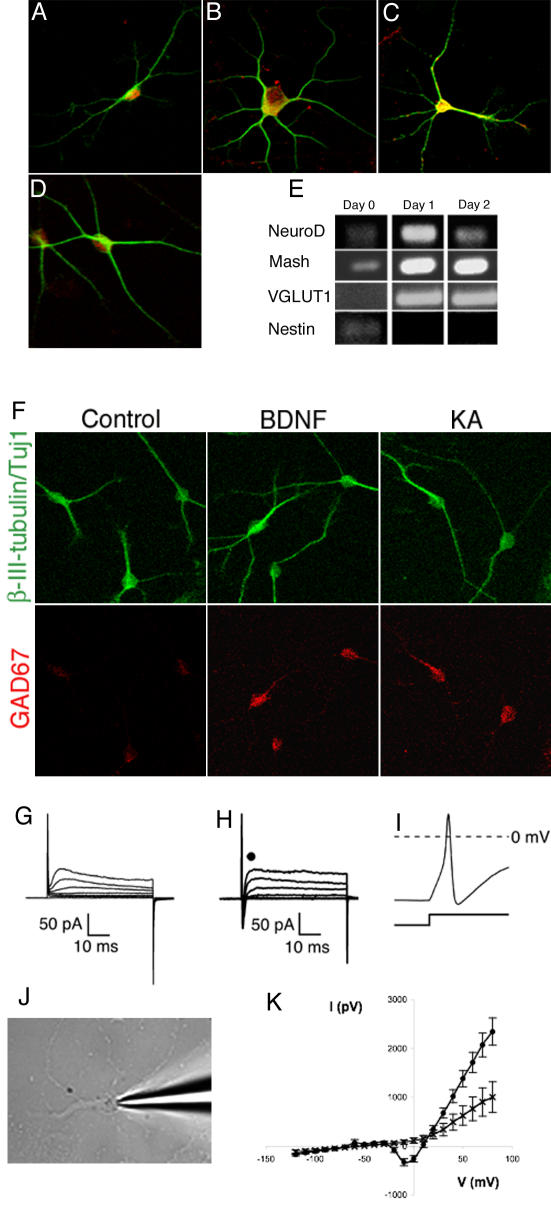
Differentiated neurons originating from hippocampal precursor cells showed features of dentate gyrus granule cells. After differentiation the cells were fixed and stained for markers characteristic of dentate gyrus granule cells. A; β-III-tubulin (green), Prox1(red). B; β-III-tubulin (green), Calbindin (Red). C; β-III-tubulin (Green), Synaptoporin (Red). D; β-III-tubulin (Green), NeuN (Red). E; Differentiation led to up-regulation of molecular neuronal markers and down-regultion of precursor cell markers. Proliferating cultures (day 0) were allowed to differentiate and mRNA was extracted after different times. Differentiation led to up-regulation of NeuroD1, mash1, VGLUT1 transcripts, whereas Nestin was down-regulated. F; Up-regulation of GAD67 by differentiated neurons. Neurons differentiated for 10–12 days were washed once with medium and subjected to a 2.5 h stimulation of kainate (KA, 10 µM) or rhBDNF (100 ng/ml) in fresh medium and subsequently fixed and evaluated. GAD67 (red) was upregulated in β-III-tubulin-positive cells (green), a property associated with granule neurons in vivo. The images were captured with identical laser settings at the confocal microscope. G; Electrical properties of cultured dentate gyrus precursor cells and differentiated neurons. Membrane properties of cultured dentate gyrus precursor cells and neurons derived from them were measured while voltage-clamped to −60mV. G, H, J, K; Typical membrane currents recorded by series of depolarizing and hyperpolarizing voltage steps ranging from −120 to+80mV, with 10-mV increments (only first 7 voltage steps are shown). G; On depolarizing steps in voltage-clamps, cells showed typical features of precursor cells with outward rectifying Potassium channels. H; differentiated neurons showed properties consistent with sodium currents. I; In current-clamp, single action potentials were elicited in differentiated neurons by injecting current pulses in 20mV-increments for 100 ms. K; Current voltage curves of the fast inward currents recorded from differentiated neurons with and without TTX were constructed. Mean±SEM values averaged from day 28 old cultures are shown (•; n = 11). Such currents were completely blocked by 1 µM TTX (n = 3).

We next asked, if the new neurons generated in vitro would reveal characteristics of their in vivo counterpart, the granule cells of the dentate gyrus. In addition to pan-neuronal marker NeuN (Fig. 3d), the differentiated neurons in culture expressed transcription factor Prox1, calcium-binding protein calbindin and synaptic vesicle protein synaptoporin, three markers characteristic of granule neurons in vivo (Fig. 3a, b, and c) [26], [27]. Prox-1 is a transcription factor specifically expressed by dentate gyrus granule cells [27]. RT-PCR revealed that NeuroD1 and Vesicular Glutamate Transporter 1 (VGLUT1) [28] were up-regulated during differentiation (Fig. 3e), whereas nestin mRNA, indicative of the precursor cell stage, was down-regulated. Proliferating cells showed no or undetectable levels of NeuroD1 and VGLUT1.

To further confirm the regional identity of the neurons generated form these precursors we exploited the ability of dentate granule neurons to express GABA along with glutamate after excitation [29], [30]. Dentate granule cells are unique neurons in that they can release both glutamate and GABA as neurotransmitters, most evidently in the hyperexcitability during seizures. We treated differentiated neurons from precursor cells with 10 µM kainic acid for 3 h and then stained with an antibody specific to GAD67, the key enzyme of GABA synthesis and responsible for GABA induction in mature granule neurons. As expected there was an up-regulation of GAD67 after kainate treatment (Fig. 3f), thus reproducing another granule cell characteristic in vitro. Addition of 100 ng/ml recombinant brain derived neurotrophic factor (BDNF), which also induces the GABAergic phenotype in granule cells in vivo [30], led to a similar upregulation of GAD67 (Fig. 3f).

To unambiguously assess neuronal identity of the differentiated cells we undertook an electrophysiological characterization. Whole cell patch clamp recording revealed that proliferating cells had patterns of membrane properties resembling the related precursor cells from dentate gyrus in vivo with outward rectifying potassium channels (Fig. 3g) [16], [31]. By 4–5 days into differentiation, the outward current persisted and the cells showed only minimal inward current. In contrast, by 20–30 days there was a prominent inward current although the outward currents persisted. The resting membrane potential of the neurons was −59±4 mV. In voltage clamp mode depolarization steps elicited initial spikes of inward current in 11 out of 20 cells. Tetrodotoxin (TTX) blocked these currents suggesting that they originated from voltage-gated sodium channels (Fig. 3 h–j). In current clamp mode the injection of currents evoked action potentials (Table 1). Similar to newborn granule cells in vivo, the neurons differentiated from precursor cells in vitro generated only single action potentials, when injected with currents in the current clamp mode [32].

**Table 1:** Electrophysiological characterization of neurons differentiated from adult dentate gyrus precursor cells.

Age	n	Input Resistance (MΩ)	Resting membrane potential (mV)	Na current Amplitude (pA)	AP width (ms)	AP threshold (mV)	AP Amplitude (mV)	AP peak (mV)
DIV 3	9/9	555±110	−64±4	n/a	n/a	n/a	n/a	n/a
DIV 28	11/15	894±202	−59±4	400±72	3.8±0.3	−24±1	37±2	13±2

### Exogenous factors influence the neuronal differentiation potential of neural precursor cells

**Figure 4:**
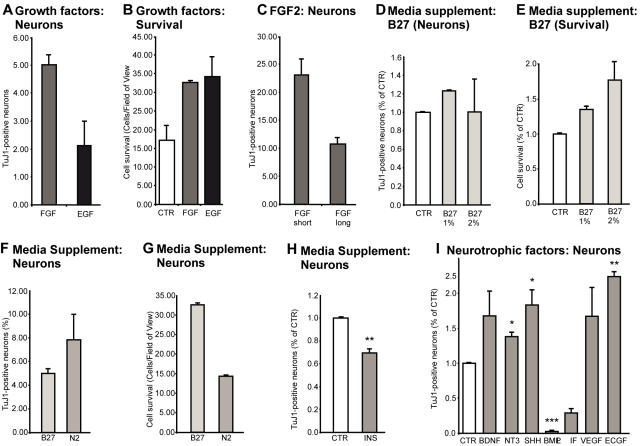
Effect of media and growth factors on precursor cells. A; FGF2 increased neuronal differentiation compared to EGF. Precursor cells differentiated in the presence of re-introduced EGF and FGF were stained with β-III-tubulin four days after differentiation. B; Both FGF2 and EGF increased cell survival in the cultured compared to control. C; FGF showed a differential effect on β-III-tubulin-positive neuronal differentiation depending on the duration of the treatment. Short-term treatment led to increased numbers of β-III-tubulin positive neurons from precursor cells, whereas prolonged FGF led to a decrease. D, E; Serum increased neuronal differentiation in a dose-dependent manner. There was also a general increase in survival with increasing concentrations of B27 supplement. F, G; B27 and N2 had complementary effects on precursor cells. B27 increased the survival of the precursor cells, whereas N2 increased neural differentiation. H; Insulin alone was not sufficient to explain the effects of B27 or N2. I; Various neurotrophic factors had pronounced and differential effects on the precursor cells. BDNF, NT-3, Shh, VEGF, ECGF caused an increase in β-III-tubulin positive neurons. LIF and BMP showed potent pro-gliogenic effects. *, p<0.05; **, p<0.005; ***, p<0.0005

Under proliferation conditions, cell density was significantly greater with EGF or FGF2 alone than without any growth factors, suggesting that both factors exerted a pro-proliferative effect, with EGF being more potent than FGF2 (Fig. 4b). We found a strong difference in neuronal differentiation between simultaneous withdrawal of EGF and FGF2 compared to sequential growth factor withdrawal. When a low concentration of FGF2 (5 ng/ml) was added to the growth factor-withdrawn precursor cells, a greater number of neurons was generated compared to a low concentration of re-added EGF (Fig. 4a). This result is similar to an earlier study suggesting a neurogenic role of FGF2 on rat hippocampal precursor cells [9]. Cell density with EGF or FGF2 alone, however, was significantly greater than without any growth factors, suggesting that both the growth factors had a pro-proliferative effect (Fig. 4b). Prolonged neural differentiation in the presence of low FGF2 (5 ng/ml) resulted in a higher cell density in the culture but led to a decrease in the number of cells that differentiated into neurons (Fig. 4c). When precursor cells were differentiated in the presence of EGF alone, the cells showed morphological features of glial cells. Taken together with the previous result that EGF together with FGF2 was required for maintaining precursor cell renewal, the most parsimonious conclusion from this finding is that EGF promotes the self renewal of precursor cells and favors glial differentiation, whereas FGF2 predominantly maintains their neurogenic potential.

We also noted differential effects of different commercially available supplements used with the culture medium. B27 did not alter neuronal differentiation (Fig. 4d) but consistently produced an increase in cell survival (Fig. 4e). N2 produced a slightly higher neural differentiation but led to decrease in the survival of cells (Fig. 4f). We thus chose B27 for all further experiments. Because one of the principle protein components in both B27 and N2 is insulin, we examined the role of insulin in neuronal differentiation in our cultures. We found that the effects of N2 and B27 could only partially be recapitulated by insulin alone (INS) (Fig. 4h), suggesting that additional factors are responsible for the net effects of N2 and B27 (Fig. 4f). As the added concentration of insulin (20 µg/ml) has been reported to induce the activation of the receptor for insulin-like growth factor (IGF), insulin signaling could be one primary mechanism of regulating neuronal differentiation [33].

Several growth and trophic factors besides EGF and FGF2 reportedly influence the differentiation of fetal or immortalized precursor cell cultures [34], [35]. To test for their effectiveness in our model we grew hippocampal precursor cells under adherent conditions and added different exogenous trophic factors while withdrawing EGF and FGF2.

Neurotrophins have a broad range of effects on neuronal maturation and survival and act through tyrosine kinase receptors (Trk). BDNF, which has been linked to structural plasticity and regulation of adult neurogenesis in vivo [36], [37], caused a robust increase in neuronal differentiation in our culture system (Fig. 4i). The neurotrophic factor neurotrophin 3 (NT3) is expressed at high levels in the dentate gyrus [38]. NT3 was the only growth factor that showed a high association with adult hippocampal neurogenesis in a screen for genes associated with adult neurogenesis in a set of recombinant inbred strains of mice [39]. In the present study, addition of NT3 significantly raised the differentiation of neurons by about 40% (Fig. 4 i). Addition of the paracrine factor sonic hedgehog (Shh) that plays numerous roles in controlling stem cell activity in the developing brain [40] and induces neuronal differentiation in cells derived from adult rat hippocampal cells [41], [40], [42], increased neuronal differentiation by about 80% (Fig. 4 i).

Bone morphogenic protein 2 (BMP2) and leukemia inhibiting factor (LIF), in contrast, showed a significant reduction in cells that differentiated into neurons (Fig. 4i). These data are consistent with the fact that BMP2 and LIF are potent anti-neurogenic in several stem cells culture conditions and induce astrocytic differentiation [43]. There effects had not yet been tested on adult hippocampal precursor cells.

In the intact adult dentate gyrus in vivo, endothelial cells are in close proximity to the precursor cells [23], [24], [25]. Fetal stem cells, when cultured together with endothelial cells, derived more neurons than when cultured alone [25]. In addition, vascular endothelial growth factor (VEGF) has shown a strong regulatory effect on adult hippocampal neurogenesis in vivo [44]. When we added two endothelia derived factors, VEGF or endothelial cell growth factor (ECGF), we found a robust increase in neuronal differentiaton (Fig. 4i).

### Single-cell analysis of precursor cells reveals self-renewing properties and multipotency

As key criterion of stemness, we studied the potential for self-renewal [7]. Self-renewal is defined operationally as the ability of individual cells to maintain precursor cells properties after division. Daughter cells of precursor cells should show the same precursor cell properties than the mother cell. As studying self renewal is technically challenging under adherent conditions, we made use of the colony-forming capabilities of the detached precursor cells [7]. At 10–14 days after isolation, the cells were trypsinized, diluted and plated at a density of 1000 cells per well (24-well plate) as previously defined to provide optimal clonal growth [4]. If there were indeed stem cells among the isolated cells, the single cells should be able to self renew and give rise to secondary colonies with some of the cells within the spheres again showing stem cells properties (tertiary colonies). These colonies resemble “neurospheres” and behave similar. The term “neurosphere,” however, is now widely used as standing for an entire paradigm in neural precursor cell biology [7], which is different from the monolayer protocol, and not just the description for the floating aggregates that form from putative precursor cells in vitro. We hence chose the term “sphere-like colony” here to avoid misunderstandings, especially in sentences that juxtapose the two paradigms. We neither intend to introduce yet another nomenclature nor to load established nomenclatures with unjustified meanings.

**Figure 5:**
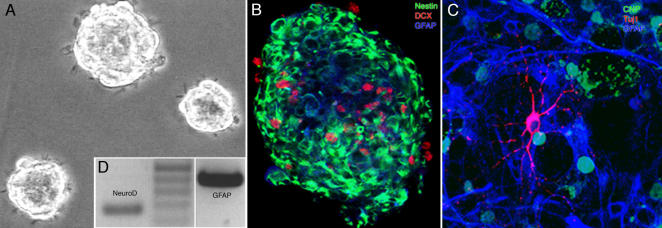
Precursor cells from dentate gyrus showed self renewal when plated in very low, so-called “clonal density” or one-per-well, suggestive of the presence of cells with stemness properties. A; Individual neural precursor cells proliferated and gave rise to neurosphere-like colonies. B; Single sphere-like colonies in proliferation conditions contained a mixture of cells at different stages of development and differentiation. Larger clusters showed a differentiated core that lacked nestin expression (Green) but was positive for astrocytic marker GFAP (Blue). Neuronal differentiation as judged by the Doublecortin (Red). In small spheres, Nestin was also found in the center of the agglomerate. C; Upon transfer into differentiation conditions, the cells differentiated into the three neural lineages: neurons (β-III-tubulin, Red), astrocytes (GFAP, Blue), oligodendrocytes (CNPase; Green). D; Under differentiation conditions, single-cell derived sphere-like colonies up-regulated both neuronal and glial genes (RT-PCR for NeuroD and GFAP).

The plated single cells began to proliferate and formed sphere-like colonies after 6 days. We scored for the number of sphere-like colonly obtained per number of precursor cells plated. After 10 days we found that 17.5±2.1% of the cells plated at “clonal density” gave rise to sphere-like colonies (Fig. 5a). This rate similar to the self renewal reported for precursor cells obtained from rat hippocampus [3]. Recent studies have suggested that even at low densities, putatively clonal neurospheres tend to fuse and give rise to chimeras [45], [46]. To unambiguously assess whether our precursor cells indeed had the ability to self-renew, we aspirated single cells under the microscope and placed them into individual wells of a multiwell plate. Formation of sphere-like colonies was assessed 6 days later and we found that 15.6% of the wells contained sphere-like colonies. Such colonies were subjected to the assay for multipotency in order to demonstrate the three-lineage differentiation potential of the individual cells (see below). Because single cells had been plated the different lineages could not originate from separate precursor cells present in the culture. To demonstrate “self-renewal” of the cells that constitute these secondary colonies it has additionally to be shown that these same colonies contain cells that upon individualization would again give rise to proliferating cells with multi-lineage potential.

To assess whether secondary colonies indeed contained self-renewing precursor cells, single secondary clusters were dispersed to obtain single cell suspensions and were plated again as described above. The plated cells again generated tertiary sphere-like colonies that showed no overt difference from the secondary colonies. After 10 to 14 days, ∼10% of the plated primary cells had generated tertiary clusters. Thus, under the assumptions and conditions of this assay, precursor cells from the adult murine dentate gyrus fulfilled the criteria of self-renewal.

We had done the above studies in C57Bl/6 mice, whereas other reports on murine neural precursor cells had used CD1 mice [4]. We wondered whether strain-related differences might contribute to the reported differences in stem cells characteristics. When we repeated our experiments with dentate gyrus precursor cells from out-bred strain CD1 we obtained identical results (data not shown).

We compared self-renewal in fresh with passaged cultures. Self-renewal could be successfully demonstrated at the 20^th^, 40^th^, and 60^th^ passage. Irrespective of the passage number the precursor cells showed immunoreactions for nestin and BrdU. These results suggested that over successive passages precursor cells from the adult dentate gyrus could self-renew.

We next assessed whether cells contained within the neurosphere-like clusters were capable of generating differentiated cells of the three neural lineages: neurons, astrocytes, and oligodendrocytes. Differentiation of the clones was studied by transferring individual clusters onto laminin-coated surface, and allowing them to differentiate for one week. Differentiation was induced by withdrawing the mitogens and adding fetal bovine serum and retinoic acid. At the time of plating, the core of the clusters was largely composed of differentiated cells, many of which stained for GFAP or doublecortin (DCX)–a marker associated with migratory neuroblasts in vivo (Fig. 5b) [47]. A total of 15 neurospheres were fixed and stained. Optical sections were analyzed under the confocal microscope to reveal the core and the periphery of the cluster. We found that the larger the size of the sphere, the less nestin expression was seen within the core. One week after plating, the spheres had flattened and showed cell morphologies suggestive of differentiation. Immunocytochemistry revealed that 75% of the secondary sphere-like colonies generated neurons (β-III-tubulin), astrocytes (GFAP) and oligodendrocytes (CNPase; Fig. 5c). We performed the same assay for the differentiation of tertiary spheres that had been generated from secondary sphere-like colonies. The tertiary colonies, too, generated neurons, astrocytes and oligodendrocytes.

Differentiating cultures up-regulated mRNA for NeuroD1, a key transcription factor in the neuronal lineage (Fig. 5d) and also expressed mRNA for GFAP. As with the self-renewing precursor cell populations, tri-lineage potential at the 20^th^, 40^th^, and 60^th^ passage could be observed as judged by the immunostaining for β-III-tubulin (neurons), GFAP (astrocytes) and CNPase (Oligodendrocytes). Again, roughly three quarters of the colonies showed the three-lineage potential and thus no appreciable decrease in neuronal differentiation with passage number.

## Discussion

We have demonstrated here that neural precursor cells with certain stemness properties could be isolated and propagated from the adult murine hippocampus and that the progeny of these cells generated neurons with phenotypes very similar to granule cells in vivo.

We describe an optimized method to reliably obtain monolayer cultures of neural precursor cells from the microdissected dentate gyrus of adult mice. The buoyancy enrichment method used in our protocol allowed us to normalize the initial plating density of the immature cell populations and thus to overcome a density dependent growth limitation that might have affected the outcome in previous studies [4], [5]. Such density dependent gradient enrichment has been used before to separate non dividing cells from the precursor cells contained in tissue from rats and humans [9], [48]. In contrast to previously described protocols, following buoyancy enrichment the neural precursor cells were maintained in a serum free environment with EGF and FGF2 as growth factors [9], [48]. The constituents of the B27 (or N2) media supplements provided the necessary factors for long-term maintenance. Precursor cells from rat hippocampus have been propagated with FGF2 alone, but mouse cells have resisted this technique. Addition of EGF was essential for long term propagation of mouse dentate gyrus precursor cells under adherent conditions.

EGF has also been used to grow precursor cells as neurospheres, a widely used strategy to culture precursor cells from the adult brain, originally described by Reynolds and Weiss in 1992 [49]. One potential problem is that neurosphere cultures tend to generate differentiated cells in their core. Consequently, the interaction between differentiating cells and precursor cells may expose the stem cells to paracrine factors that promote differentiation [7]. The adherent culture system overcomes these problems by allowing the cells to remain more isolated and to be continuously nurtured by the growth factors in the medium, thereby maintaining a higher degree of homogeneity. This greater homogeneity does not imply that in monolayer cultures all cells would be identical or all cells would be precursor cells. Spontaneous differentiation, however, could be minimized.

Studies in the developing telencephalon have suggested that radial glia can function as stem cells that generate cortical neurons [50], [51], [52], [53]. Similarly, a subset of proliferating cells in the SGZ of the adult dentate gyrus have an antigenic and morphological profile similar to radial glia. It was found that these cells, too, act as the local precursor cells in vivo [54], [19]. Our present data suggest that cells with radial glia-like properties can also be isolated from the adult dentate gyrus. We have previously reported that the population of transient amplifying progenitor cells in vivo (type-2 cells) retains some antigenic features of radial glia cells, although they do not show a radial morphology [19]. We do not claim that our culture protocol specifically allows the propagation of hippocampal radial glia. But our results indicate that dentate gyrus precursor cells ex vivo retain properties of their putative in vivo counterparts.

Many studies interested in neural precursor cells have taken a bio-engineering approach and focused on strategies to derive dopaminergic or serotonergic neurons for transplantation. These phenotypes, however, lie outside the physiologic range of dentate gyrus precursor cells. To us it seems necessary to first understand the intrinsic potential of the precursor cells before attempting to divert their developmental path.

The mechanisms of regional specification of hippocampal precursor cells has not been analyzed in greater detail, except for a few reports that aimed at delineating broader differences between precursor cells in the SGZ and the SVZ [4], [5]. The molecular program underlying granule cell development is not yet fully understood. With Prox1, however, one highly specific transcription factor for granule cell differentiation is known [55]. We here could show that the differentiating neurons in our cultures expressed Prox1.

There has been some controversy regarding the presence or absence of neural “stem cells” (in the stricter sense of the definition) in the SGZ in contrast to the SVZ [3], [4], [5]. We cannot finally settle this issue. Our dissection protocol, however, certainly minimized any risk for contamination with cells from the SVZ of the lateral ventricles (Fig. 1). Admittedly, to avoid contamination with cells from the walls of the 3^rd^ ventricle is much more difficult but was painfully attempted by using thin tissue sections and placing a clear cut between the ventricular wall and the granule cell layer (Fig. 1d). Given the very low proliferative activity in the ventricular wall of the 3^rd^ ventricle [11] the risk of mistaking 3^rd^ ventricle precursor cells as hippocampal was further diminished.

In summary, based on our present data we propose that with regard to the existence of precursor cells the differences between dentate gyrus and SVZ are not qualitative but rather quantitative. Previous data from rat models and our experience in the present set of experiments indicate that the abundance of stem-like cells in the dentate gyrus is significantly lower than in the SVZ [22]. The cellular density of the neurogenic zone is more compact in the SGZ than in the SVZ. The SVZ is flanked by the ventricular lumen, whereas the SGZ is surrounded by tissue with an extensive neuropil. Hence any enzymatic digestion of the SGZ leads to damage of cellular processes, which might explain the reduced recovery of surviving cells from the SGZ. In addition, our dissection method included the medial tip of the dentate gyrus, which was excluded in an earlier study that could not detect cells with stem cell-like properties in the SGZ. The tip area contains the highest density of proliferating precursor cells in vivo [56]. Again, we use the term “stem-like cells” here in order to relate our data to the published discussion, which spoke plainly of “stem cells”. We suggest that the hippocampal precursor cells in our cultures showed more stemness properties than stated by these previous publications. At the same time the definition of “stem cells” remains notoriously difficult. To avoid misunderstandings we prefer the more generic term “precursor cells”.

Our protocol is based on established methods [22], [3], [4], [7] but substantial modifications to the available rat protocols had to be introduced (Table 2). These modifications combined the gradient enrichment procedure originally described for rat hippocampal precursor cells with the addition of both EGF and FGF2, the use of B27 as the serum supplement, the control of appropriate plating densities, and the optional use of additional exogenous factors for the trophic support of the cultured cells. EGF and FGF2 together with B27 serum supplement suppressed differentiation and maintained the precursor cells at a stem cell stage. We noted that EGF is mandatory for maintaining stemness properties of precursor cells isolated form the adult murine dentate gyrus. This is in agreement with previous studies in vivo that suggested an increase in proliferation after infusion of EGF [57] compared to FGF2. In that study neuronal differentiation was more pronounced after infusion of FGF2 than of EGF. Similarly we found that EGF had a potent pro-proliferative effect on the precursor cells in vitro, whereas FGF maintained their neurogenic potential.

**Table 2:** Comparison of precursor cell culture protocols from the adult hippocampus

	Present study	Palmer et al., 1997 [3]	Palmer et al., 1999 [9]	Seaberg et al., 2002 [4]	Roy et al., 2000 [63]	Bull et al., 2005 [5]
**Species**	Mouse	Rat	Rat	Mouse	Human	Mouse
**Type**	Monolayer	Monolayer	Monolayer	Neurospheres	Monolayer	Neurospheres
**Region**	Subdissected dentate gyrus, including tip	Hippocampus	Hippocampus	Subdissected dentate gyrus, excluding tip	Dentate Gyrus	Dentate Gyrus
**Enrichment proc.**	Percoll	–	Percoll	–	Promoter based FACS	Promoter based FACS
**Plating density**	10^4^ cells/cm^2^	No information	No information	20cells/µl	0.1ml/35mm dish of 10^7^cells/ml of cell suspension	7000cells/cm^2^
**Media**	Neurobasal	DMEM-F12	DMEM-F12	DMEM-F12	DMEM-F12	NeuroCult
**Supplement**	B27	N2	N2	N2/B27	N2, Platelet-depleted Bovine Serum	NeuroCult NSC supplement with 2% BSA
**Growth factor**	EGF, FGF2	FGF2	FGF2	EGF, FGF2, Heparin	FGF2	EGF, FGF2, Heparin, BDNF
**Coating**	PDL, Laminin	Polyornithine, Laminin	Polyornithine, Laminin	–	Laminin	–
**Test for stemness**	17.5±2.1%	–	21%	0.54±0.1 per 10,000 cells	–	0
**Max. passage no.**	60	–	–	–	–	3
**Functional assay**	Electrophysiology, GAD induction	–	–	–	Electrophysiology	–

Precursor cells in the adult SGZ are located in a privileged niche with a distinct cellular composition that controls stem cell activity [58], [25]. The mediating niche factors have been of intense research activity. Some of these molecules have been previously implicated in decisive developmental patterning of the nervous system. The precursor cells were highly susceptible to the presence of EGF. Among all factors studied here EGF produced the most potent effect on proliferation. Morphogen Shh strongly increased neural differentiation from the precursor cells. Other reports had found similar effects both in vitro and in vivo [41], [40], [42]. Factors released by endothelial cells such as VEGF biased the precursor cells to acquire a neuronal phenotype, whereas BMPs and LIF were pro-gliogenic in our culture system and reduced neuronal differentiation (Fig. 4i). BMPs have previously been shown to produce a gliogenic niche for the SVZ precursor cells [59]. The result that multiple factors differentially regulate precursor cell differentiation suggests that these factors might work in concert to guide neurogenesis from the precursors cells stage to final functional integration in vivo.

That our cell culture protocol can mimic a sufficient number of in vivo parameters is also underscored by the fact that the differentiated neurons fulfilled the key criteria of neuronal functionality: they generated sodium currents and fired action potentials. We noticed that during maturation the inward currents increased, whereas the outward currents (possibly K^+^) showed little change. This suggests that outwards currents may be required in the transition from immature to mature stages. The hyperpolarizing currents may prevent potentially damaging massive Ca^2+^entry from sustained depolarization.

Besides growth factor effects on functional maturation it is likely that precursor cells are driven towards differentiation by the neurons that make synaptic contact with them. This effect might again be based on the secretion of soluble factors or on direct effects of neurotransmitters. For example, GABAergic input is one of the earliest input received by the newborn neurons [31], [60]. GABA has excitatory effects on the immature cells and promotes further differentiation [61], [60]. In addition, some of the factors with pro-neurogenic effects (such as NT-3 and Shh) can be secreted by GABAergic interneurons [62]. This suggests that GABA might play a key role in regulating adult hippocampal neurogenesis by controlling levels of network activity and the release of morphogens in the vicinity of precursor cells. Our cell culture also responded to neurotrophic factors in a way predictable from the in vivo situation, including the characteristic induction of a GABA-like phenotype in granule cells in response to BDNF.

We here report a modified method to isolate and propagate neural precursor cells from adult murine dentate gyrus as adherent monolayer culture system. Mouse dentate gyrus precursor cells had the capability to self-renew in a single-cell assay and differentiate into neurons, astrocytes and oligodendrocytes. The precursor cells expanded in vitro showed plasticity towards acquisition of neuronal phenotypes depending on the culture conditions. In many regards the neurons derived in our cultures corresponded to their in vivo counterpart, the granule cells of the dentate gyrus. These precursor cells culture from which granule cells can be generated will provide a useful research platform to study the regulatory mechanisms of adult hippocampal neurogenesis in mice.

## Materials and Methods

### Tissue Dissection

All institutional regulation regarding animal ethics was followed. Adult female animals (C57Bl/6 or CD1) were killed by an overdose of ketamine and decapitated. The brains were removed from the skull and were placed in cold artificial CSF (aCSF) that was constantly bubbled with 95%O_2_/5% CO_2_. The aCSF consisted of the following ingredients (in mM): NaCl, 124; KCl, 2.5; NaH_2_PO_4_, 1.25; CaCl_2_, 1; MgCl_2_, 1; NaHCO_3_, 25; D-Glucose, 10. The brains were sliced coronally (300 µm) using a vibratome and the coronal slices with the hippocampus and the dentate gyrus collected and placed in cold aCSF. The slices were then moved under a dissecting microscope to dissect out the dentate gyrus and free it from the hippocampus and the surrounding ventricular tissue (Fig. 1). This was done by drawing a wedge along the hippocampal fissure separating the dentate gyrus from the rest of the hippocampus. A cut was then place between the dentate gyrus and the ventricular surface on one side and the CA3 region on the other. The dissected dentate gyrus was kept in aCSF till further procedures were carried out. The dissected dentate gyri from 4–5 animals per preparation were pooled.

### Tissue digestion

The pooled tissue was dissociated by digestion with a mixture of Papain (Worthington), Dispase (Roche) and Deoxyribonuclease (Worthington) for 30 to 40 min. The enzymes were removed by washing twice in Phosphate Buffered Saline (PBS). The cell mixture was passed through a 40 µm cell strainer (Becton Dickinson) to obtain a single-cell suspension. To assess precursor cells enrichment, cell mixture underwent centrifugation at 20,000×g for 30 min in a continuous Percoll gradient. The stock of isotonic Percoll (Amersham) was prepared by diluting nine parts of Percoll with one part 10X PBS. After gradient separation the cells were washed and transferred to proliferation medium. Based on the sedimentation of the mouse cells in the continuous gradient we determined that precursor cells could be selectively enriched in the cell pellet formed in a solution of 22% Percoll following low speed centrifugation (500–1000×g, 10 minutes, room temperature). A common and simple procedure now used in our laboratory is to enrich a tissue preparation for precursor cells using an initial low-speed separation in 20–35% Percoll (empirically determined for each strain and species). The precursor cells and erythrocytes in the pellet are collected and then resuspended in a mixture of 65–70% Percoll (also empirically determined). The erythrocyte pellet is discarded and floating precursor cells are rinsed free plated into growth medium.

### Cell culture

The surface of the culture dishes (polystyrene Petri dishes and culture flasks, or glass coverslips in multiwell plates; Nunc) was first coated with 10 µg/ml Poly-Lysine (Sigma) overnight at room temperature. After several rinses in water, the surfaces were then coated with 5 µg/ml Laminin (Tebu-bio&Roche) at 37°C overnight. The plates or coverslips were stored at −20°C.

After removing excess coating solution, cells were plated directly onto the surface. The cultures were maintained in proliferation medium consisting of Neurobasal (Invitrogen), B27 or N2 supplement (Invitrogen), 2 mM Glutamax (Invitrogen), Pen-Strep (Sigma), 20 ng/ml human Fibroblast Growth Factor-2 (FGF2), and 20 ng/ml human Epidermal Growth Factor (EGF) (both from R&D or Pepro Tech). The medium was replaced with fresh medium the next day. Subsequently, the cultures were fed with new medium every two or three days by replacing 75% of the medium. We maintained a cell density of 10^4 ^cells/cm^2^. To induce and maintain differentiation the growth medium was replaced with either growth medium free of mitogens FGF2 and EGF, with or without 0.5 µM retinoic acid (Sigma), or with 0.5% fetal bovine serum and human Brain Derived Neurotrophic Factor (BDNF; 100 ng/ml). Recombinant human Insulin (Sigma) was used at a concentration of 20 µg/ml.

### Test for self-renewal and multipotency

For self renewal experiments, cells were trypsinized, triturated, centrifuged and resuspended in medium. Cells were then either plated at a very low density of (often referred to as “clonal density”) or individually aspirated under microscopic control and seeded in separate wells of a microtiter plate. The presence of only single cells per well was confirmed under the microscope 24 h later. In either case, the cells were plated in conditioned medium together with fresh medium in 1∶1 ratio with 20 ng/ml of both EGF and FGF2. The conditioned medium was prepared from medium incubated for 48 hrs in proliferating precursor cells. The media was centrifuged and the supernatant preserved at 4°C when used on the same or the next day or at −80°C for long term storage.

### Immunocytochemistry

Cultures were fixed with cooled 4% Paraformaldehyde in 0.1M phosphate buffer (pH 7.4) for 20–30 minutes. After washes with Tris buffered saline (TBS) cells were blocked with 3% Donkey serum (Chemicon) containing 0.2% Triton X-100. Primary antibodies were diluted in blocking buffer and the cells were incubated overnight. After washes with TBS, secondary antibody was diluted in TBS and the cells were incubated for 2 h at room temperature.

The following primary antibody and dilutions were used. Monoclonal: anti-Nestin 1∶400 (BD pharmingen); anti-RC2 1∶50 (DHSB Iowa); anti-β-III-tubulin 1∶1000 (Promega); anti-GAD67 1∶1000 (Chemicon); anti-NeuN 1∶100 (Chemicon); anti-Map2ab 1∶500 (Sigma); anti-CNPase 1∶100; rat anti-BrdU 1∶500 (Acccurate). Polyclonal: rabbit anti-β-III-tubulin (TuJ1) 1∶1000 (Covance); guinea pig anti-GFAP 1∶1000 (Advanced Immunochemistry); goat anti-Calbindin 1∶250 (SWANT); rabbit anti-Prox-1 1∶5000 (Chemicon); rabbit anti-Synaptoporin 1∶200 (Chemicon); rabbit anti-BLBP 1∶2000 (kind gift of Nathaniel Heintz, Rockefeller University); Goat anti-DCX 1∶300 (Santa Cruz). Fluorescence-coupled secondary antibodies were raised in donkey (Dianova) and used at 1∶250.

A Leica TCS/SP2 confocal microscope (Leica, Bensheim) was used to evaluate fluorescent stainings using appropriate excitation beams and emission filters. All analyses were done in sequential scanning mode to avoid channel bleeding and the detection of spurious double-labeling. Counting was done with a Zeiss Axioplan2 epifluorescence microscope with appropriate filters.

### Induction of GABA phenotype

Proliferating precursor cells were plated at 1–2×10^4^ cells/cm^2^. The cells were withdrawn from the proliferation medium and allowed to differentiate for two to three weeks. On the day of the experiment, 10 µM Kainic acid (KA) (Sigma) or 100 ng/ml of BDNF (Peprotech) was added directly to the medium and the cultures were left in the incubator for 30 min. The medium was removed and the cultures were washed twice with fresh medium. They were then incubated in fresh medium for three hours. Cells were fixed and underwent immunocytochemical procedures as described above, except that 0.25% Triton X-100 was added to the blocking solution and avoided thereafter.

### RT-PCR

Precursor cells were cultured as adherent cultures and differentiated for 5–8 days. RNA was extracted with RNAeasy kit (Quiagen). After spectrophotometric quantification, RNA from each time point was reverse-transcribed in a final volume of 50 µl. 1 µg of the RNA was mixed with 1 µg of Random Primers (Invitrogen) at 70°C for 10 min. Samples were cooled on ice and 5X RT-Buffer, 0.1M DTT, and dNTPs were added. After addition of reverse transcriptase the reaction mixture was incubated for 90 minutes at 42°C followed by 70°C for 10 minutes. Two µl of the reaction mixture was used for the PCR reaction. The annealing time was 57°C for all primers. The PCR protocol consisted of the following reaction cycles: initial denaturing at 95°C for 10 minutes followed by 40 cycles of 45s at 95°C for, 45s at 57°C, and 1 min at 72°C. The reaction was concluded by a final extension of 10 minutes at 72°C. Products were run on 1% agarose gels and analyzed. Equal volume of cDNA was added for comparison. The primers are listed in Table 3.

**Table 3:** PCR primer sequences

Nestin	forward	5′–AGGTTTGAAGACGCAGAGGA–3′
	reverse	5′–TTCGAGAGATTCGAGGGAGA–3′
GFAP	forward	5′–CACGAACGAGTCCCTAGAGC–3′
	reverse	5′–TCACATCACCACGTCCTTGT–3′
Pax6	forward	5′–ACAGAGTTCTTCGCAACCTGGCTA–3′
	reverse	5′–ACTGGTACTGAAGCTGCTGCTGAT–3′
BLBP	forward	5′–TTCGGTTGGATGGAGACAAGCTCA–3′
	reverse	5′–GCTTCATTAGCTGGCTAACTCTGGGA–3′
Vimentin	forward	5′–AGATGGCTCGTCACCTTCGTGAAT–3′
	reverse	5′–TCCTTCTTGCTGGTACTGCACTGT–3′
NeuroD1	forward	5′–ATTGCGTTGCCTTAGCACTT–3′
	reverse	5′–TGCATTTCGGTTTTCATCCT–3′
Sox2	forward	5′–CTCTGCACATGAAGGAGCAC–3′
	reverse	5′–ATGTAGGTCTGCGAGCTGGT–3′

### Electrophysiology

Patch electrodes with a resistance of 4-7MΩ were pulled from borosilicate capillaries using a laser-based pipette puller (Sutter Instrument, Novato, USA). The extracellular solution (pH 7.3) consisted of : 5mM Hepes, 150mM NaCl, 5.4mM KCl, 1mM MgCl_2_, 2mM CaCl_2_, 10mM D-Glucose. All experiments were carried out at Room Temperature. The pipette solution consisted of 130mM KCl, 2mM MgCl_2_, 0.5mM CaCl_2_, 2mM Na-ATP, 5mM EGTA, 10mM Hepes. The pH was adjusted to 7.3 with KOH. Whole cell voltage clamped recordings were obtained using as EPC 9/2 patch clamp amplifier together with TIDA software (HEKA Germany).
